# Single‐cell RNA‐seq reveals the invasive trajectory and molecular cascades underlying glioblastoma progression

**DOI:** 10.1002/1878-0261.12569

**Published:** 2019-09-17

**Authors:** Bo Pang, Jinyuan Xu, Jing Hu, Fenghua Guo, Linyun Wan, Mingjiang Cheng, Lin Pang

**Affiliations:** ^1^ College of Bioinformatics Science and Technology Harbin Medical University China

**Keywords:** glioblastoma, glioblastoma stem cells, invasion, single‐cell RNA sequencing, stem‐to‐invasion path

## Abstract

Glioblastoma (GBM) is the most common and aggressive primary brain tumor, in which GBM stem cells (GSCs) were identified to contribute to aggressive phenotypes and poor prognosis. Yet, how GSCs progress to invasive cells remains largely unexplored. Here, we revealed the cell subpopulations with distinct functional status and the existence of cells with high invasive potential within heterogeneous primary GBM tumors. We reconstructed a branched trajectory by pseudotemporal ordering of single tumor cells, in which the root showed GSC‐like phenotype while the end displayed high invasive activity. Thus, we further determined a path along which GSCs gradually transformed to invasive cells, called the ‘stem‐to‐invasion path’. Along this path, cells showed incremental expression of GBM invasion‐associated signatures and diminishing expression of GBM stem cell markers. These findings were validated in an independent single‐cell data set of GBM. Through analyzing the molecular cascades underlying the path, we identify crucial factors controlling the attainment of invasive potential of tumor cells, including transcription factors and long noncoding RNAs. Our work provides novel insights into GBM progression, especially the attainment of invasive potential in primary tumor cells, and supports the cancer stem cell model, with valuable implications for GBM therapy.

## Graphical abstract

Glioblastoma (GBM) is the most common and aggressive primary brain tumor with poor prognosis. Here, through reconstructing a branched trajectory by pseudotemporal ordering of single tumor cells, we determine the ‘stem‐to‐invasion path’, along which cells show incremental invasive potential and diminishing expression of GBM stem cell markers. We further identify crucial factors controlling the attainment of invasive potential of tumor cells.

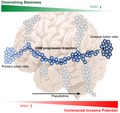

**Abbreviations**
CNV: copy number variation
CSCs: cancer stem cells
EMT: epithelial–mesenchymal transition
GBM: glioblastoma
GSCs: glioblastoma stem cells
HMM: hidden Markov model
lncRNAs: long noncoding RNAs
MSigDB: the molecular signatures database
scRNA‐seq: single‐cell RNA sequencing
TFs: transcription factors

## 1 Introduction

Glioblastoma (GBM) is the most common and highly aggressive primary tumor of the central nervous system with extremely poor prognosis despite intensive treatment (Murray *et al.*, 2014). GBM characteristically exhibits aggressive proliferation and highly invasive properties with high mortality (Kleber *et al.*, 2008). It can spread to spinal cord and other parts of the brain, which presents huge challenges to the current therapies. The dismal clinical status of GBM treatment urgently needs novel understanding of molecular mechanisms of disease progression.

In the traditional model, invasion and metastasis were the late events during tumor development and progression, which is considered to be attributed to cancer stem cells (CSCs) by many studies (Jaraiz‐Rodriguez *et al.*, 2017; Liu *et al.*, 2015; Miao *et al.*, 2015). However, increasing evidence has shown that epithelial–mesenchymal transition (EMT) and dissemination can actually occur early during cancer progression (Eyles *et al.*, 2010; Hosseini *et al.*, 2016; Linde *et al.*, 2018; Rhim *et al.*, 2012). These studies suggest that cells may acquire the invasive and metastatic ability in primary tumors, which have been ready to disseminate to distant sites and also contribute to the high heterogeneity of tumors especially GBM (Meyer *et al.*, 2015). Thus, detailed investigation of these processes can reveal the important events during GBM progression and identify the molecular determinants of invasion.

Although traditional bulk tumor analyses have identified key genes and pathways that drive invasion of GBM cells (Galavotti *et al.*, 2013), they provide limited insight into molecular mechanisms underlying GBM invasion. Instead, single‐cell RNA sequencing (scRNA‐seq) generates gene expression profiles at the resolution of an individual cell (Tang *et al.*, 2009), which has been comprehensively applied to reveal the heterogeneity of diverse cancers (Chung *et al.*, 2017; Li *et al.*, 2017; Patel *et al.*, 2014). It provides an unprecedented chance to capture and subtly dissect the molecular cascades, and to determine the key molecular determinants during the acquisition of invasive potential for GBM cells, similar to its application to studies on differentiation and development (Deng *et al.*, 2014; Trapnell *et al.*, 2014).

In this study, we took advantage of scRNA‐seq data to provide a detailed analysis of cellular heterogeneity, reveal the invasion‐associated progression path through which cells gradually acquire the invasive potential, and identify key factors involved in GBM progression.

## 2 Materials and methods

### 2.1 Single‐cell RNA‐seq data preprocessing

#### 2.1.1 Quantification and quality control

The raw data for most of analysis in this study were downloaded from GEO database (http://www.ncbi.nlm.nih.gov/geo/query/acc.cgi?acc=GSE57872). These data were published by Patel *et al. *(2014) and included 576 cells from five primary GBM patients (MGH26, MGH28, MGH29, MGH30, and MGH31). Reads were mapped to the reference transcriptome by bowtie (version 1.1.1) (Langmead *et al.*, 2009), and gene expression levels were quantified as transcripts per million (TPM) using rsem (version 1.2.28) (Li and Dewey, 2011) with the option estimate‐rspd and default parameters. We excluded low‐quality cells based on two quality measures: the number of aligned reads < 2e5 or number of genes detected < 3000. In the subsequent analysis, we excluded all cells of MGH31 for their relatively lower percentages of mappable reads.

#### 2.1.2 Screen tumor cells by copy number alterations

We inferred copy number variations (CNVs) for each cell as previously described (Patel *et al.*, 2014). Briefly, all genes were ordered by their chromosomal location, and the copy number of each gene was calculated as the sliding average of log2‐transformed TPM values with a window of 100 flanking genes within each chromosome, which was then centered across all cells. Similarly, we inferred a CNV vector using normal brain data from GTEx portal (GTEx Consortium, 2013) as a control. Then, we performed hierarchical clustering and removed the nontumor cells which showed few CNVs, similar to the control. Finally, we remained 350 tumor cells.

#### 2.1.3 Normalization

For each of the remaining four patients, we identified the genes that were not expressed in at least 95% of cells for that respective patient, and removed their intersection. A total of 14 919 genes remained.

Then, we followed the normalization steps as previously described (Karaayvaz *et al.*, 2018). Briefly, we first used the Census algorithm (Qiu *et al.*, 2017) to transform the TPM values into relative counts which were negative binomially distributed. We performed this step by function relative2abs from the r package Monocle (Qiu *et al.*, 2017). Then, scran (Lun *et al.*, 2016) was used to normalize the Census counts with cell‐specific scaling factors. The r package scran specifically considers the high dropout rate of scRNA‐seq and divides the expression of each cell by the scaling factors. In this step, three cells were removed as their low transcriptomic diversity, resulting in 347 cells for subsequent analysis. Finally, we removed additional sources of unwanted variation with RUVSeq (Risso *et al.*, 2014). RUVSeq uses a generalized linear model to regress out the variation estimated from the expression of the housekeeping genes. We used a list of 98 housekeeping genes compiled by Tirosh *et al. *(2016a) (Table S1). We performed this step by function RUVg with parameter *k* = 1, which was not performed when processing lncRNA expression profiles, since there were no long noncoding RNAs (lncRNAs) which showed constant expression levels among samples like housekeeping genes.

#### 2.1.4 Extra data preprocessing

The raw data (http://www.ncbi.nlm.nih.gov/geo/query/acc.cgi?acc=GSE84465) for validation were downloaded from GEO database, which contains 3589 cells. These data were published by Darmanis *et al. *(2017) and preprocessed with the same steps as the data from Patel *et al*. Finally, 882 tumor cells remained for subsequent analysis. We also obtained the oligodendroglioma data (http://www.ncbi.nlm.nih.gov/geo/query/acc.cgi?acc=GSE70630) published by Tirosh *et al. *(2016b) and a set of single‐cell data of the normal brain (http://www.ncbi.nlm.nih.gov/geo/query/acc.cgi?acc=GSE67835) published by Darmanis *et al. *(2015), in which 4044 and 277 cells, respectively, remained after the similar filtration.

### 2.2 Clustering of tumor cells

Tumor cells were clustered using the method developed by Monocle with regressing out the patient effect. Based on the assumption that the expressions of one gene in cells of the same group fit the same linear model, we used the least square to fit a linear model for each gene in the cells of each patient. Therefore, the patient‐specific effects could be measured by the differences between linear models of different patients. Given the expression matrix and patient labels of each cell, the function lmFit in r package limma (Ritchie *et al.*, 2015) could be used to calculate the differences between fitted linear models (coefficients matrix), which was then subtracted from original expression matrix to remove the patient effect on gene expression. In this study, we followed the approach used by Karaayvaz *et al. *(2018) and did the regression of the patient effect via the function reduceDimension in the r package Monocle, which actually used the lmFit function. Genes with mean expression > 0.1 and high dispersion were used for clustering. We clustered cells by the function clusterCells in R package Monocle with parameters rho_threshold = 2 and delta_threshold = 4. Monocle uses a density‐based approach (Rodriguez and Laio, 2014) to cluster cells based on each cell's local density (rho_threshold) and the nearest distance (delta_threshold) to another cell with higher distance and automatically determine the number of clusters. Any cell with a higher local density and distance than the thresholds is considered as the density peaks, which are then used to define the clusters for all cells. We finally identified six clusters in the data from Patel *et al*. and 12 clusters in the data from Darmanis *et al*.

### 2.3 Differential expression analysis and functional annotation

We used the scde software package (version 2.2.0) (Kharchenko *et al.*, 2014) to identify the significantly highly expressed genes in each cell cluster. Briefly, this probabilistic method takes raw count data as input and fits cell‐specific error models to estimate the posterior probability of expression magnitude for a gene in each cell. Then differential expression analysis was performed using the joint posterior probability of expression in each cell cluster. We considered the genes with absolute cZ more than 2.58 (*P* < 0.01) as the significantly differentially expressed genes. Then, the functional annotations for these genes were implemented by the Molecular Signatures Database (MSigDB) (Liberzon *et al.*, 2011; Subramanian *et al.*, 2005) with hypergeometric test and the threshold of FDR < 0.05.

### 2.4 Estimation of activity for diverse signatures and pathways

GSVA algorithm (Hanzelmann *et al.*, 2013) was implemented to evaluate the relative activation status for a signature or pathway. GSVA scores for cancer hallmark and glioma‐related signatures as well as signaling pathways were calculated using predefined gene sets extracted from the MSigDB. For G1/S, G2/M, CSC, and invasive scores, we calculated the mean expression levels of that respective gene set. Gene sets reflecting the expression program of the G1/S and G2/M phases of the cell cycle were taken from Tirosh *et al. *(2016a). And here, we used data‐derived thresholds of 3 median absolute deviations above the median to define 45 cycling cells and 302 noncycling cells. CSC and invasive signatures were manually extracted from previous studies (Table S1).

### 2.5 Single‐cell trajectory reconstruction and analysis

Single‐cell pseudotime trajectories were constructed with monocle (version 2.6.4) (Qiu *et al.*, 2017). Briefly, we first selected a set of ordering genes which showed differential expression between clusters. Then, Monocle uses reversed graph embedding, a machine learning technique to learn a parsimonious principal graph, reduces the given high‐dimensional expression profiles to a low‐dimensional space. Single cells are projected onto this space and ordered into a trajectory with branch points. As called in Monocle, cells in the same segment of the trajectory have the same ‘state’. Branched expression analysis modeling was used to further test for branch‐dependent gene expression.

### 2.6 Gene expression states by HMM

We used an hidden Markov model (HMM) to predict gene expression states (on or off) throughout pseudotime as described elsewhere (Shin *et al.*, 2015). Briefly, we divided pseudotime into 20 bins, in which cells have identical states for most genes. We calculated the mean expression level in each bin as the observed variables for HMM. Then, a Baum–Welch algorithm was used to extract the most likely emission matrix and transition probability. Finally, the Viterbi algorithm used the observed variables along with output from the Baum–Welch algorithm to predict binary gene expression states.

### 2.7 Cell lines and cell culture

Human GBM cell lines U87, U251, LN229 were obtained from Shanghai Cell Bank of the Chinese Academy of Sciences (Shanghai, China). All cells were routinely cultured in Dulbecco's modified Eagle's medium supplemented with 10% FBS (PAA Laboratories GmbH, Pasching, Austria) at 37 °C in humidified atmosphere of 5% CO_2_ in air.

### 2.8 RNA interference

Short‐interfering RNAs (siRNAs) specifically against *EPAS1* were purchased from RiboBio (Guangzhou, China) and then transfected into GBM cells using Lipofectamine 2000 reagent (Invitrogen, Shanghai, China) according to the manufacturer’s protocol. Cells transfected with corresponding scrambled siRNA were used as controls. The gene silencing effect was measured by Western blotting 48 h post‐transfection.

### 2.9 Western blotting

Proteins were extracted from cell lysates with RIPA buffer (Thermo Fisher Scientific, Waltham, MA, USA) and were separated by 10% SDS/PAGE and then transferred onto PVDF membranes (Millipore, Billerica, MA, USA). Immunoblots were blocked with 5% BSA in TBS/Tween‐20 and incubated with primary antibodies overnight at 4 °C. The following primary antibodies were used: β‐catenin (Proteintech, Wuhan, China) and EPAS1 (Affinity Biosciences, Cincinnati, OH, USA).

### 2.10 Cell invasion and migration assays

Invasion and migration assays were performed using Corning chambers (Corning, Tewksbury, MA, USA) with Matrigel (for invasion assay) or without Matrigel (for migration assay) following the manufacturer’s protocol. The cells were suspended in media containing 2% FBS and were seeded on upper chambers, while media containing 20% FBS was placed in the lower chambers. After incubation for 24 or 48 h at 37 °C, the remaining cells on the upper surface were gently removed by a cotton swab. Then, cells that had invaded or migrated to the lower surface of the membrane were fixed with methanol and stained with hematoxylin and eosin. Cells in three randomly visual fields (at 100× magnification) were counted. Both experiments were repeated in triplicate independently.

## 3 Results

### 3.1 Cellular heterogeneity within glioblastoma

**Figure 1:**
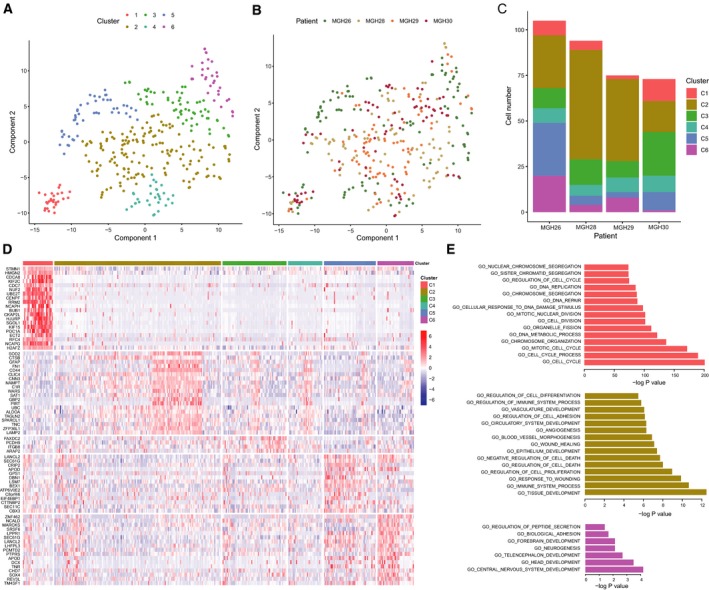
The cell clusters identified in data from Patel *et al.* (2014) (A) T‐SNE plot of tumor cells showing six clusters, in which patient effects have been regressed out. (B) T‐SNE plot showing the distribution of the patients matching (A). (C) The cell numbers of each cluster in each patient. (D) Heatmap depicting the expression of top upregulated genes in each cluster identified by SCDE. No such genes have been identified for cluster 4. (E) Functional annotations by MSigDB for genes highly expressed in clusters 1, 2, and 6. The colors are the same as those for cell clusters.

Tumor heterogeneity contributes to cancer progression and therapy failure (Kreso and Dick, 2014). We initially downloaded single‐cell RNA‐seq data from five GBM patients (published by Patel *et al. *(2014) in order to delineate the cellular heterogeneity. After stringent quality control and normalization, we analyzed a total of 347 cells from four patients (see Materials and methods). Then, we clustered all the tumor cells through excluding patient‐specific effects with linear regression (see Materials and methods). We identified six clusters of cells, all of which derived from all four patients (Fig. 1A‐C and Fig. S1A). Cluster 2 was represented by a substantial proportion of cells, which was prominent in patients MGH28 and MGH29. Cluster 3 was prominent in patient MGH30 which lacked cluster 1 cells, while clusters 5 and 6 were most prominent in patient MGH26.

We next sought to investigate the common biology of cells in each cluster through identifying cluster‐specific genes using a Bayesian method SCDE (Kharchenko *et al.*, 2014) (Fig. 1D). Except for cluster 4, we identified different numbers of significantly upregulated genes in clusters ranging from 4 to 336 (Table S2). Functional enrichment analysis of these genes revealed significant enrichment for cell cycle processes in cluster 1 (Fig. 1E). Genes specific in cluster 2 were involved in cell adhesion, response to stress, and development, while upregulated genes in cluster 6 were mainly related to brain development such as central nervous system development, forebrain, and telencephalon development. We did not identify functional annotations for clusters 3, 4, and 5 due to the small number of genes. These findings implied the existence of cell subpopulations with high heterogeneity in GBM.

### 3.2 Cell clusters reflect diverse tumor‐related status

**Figure 2:**
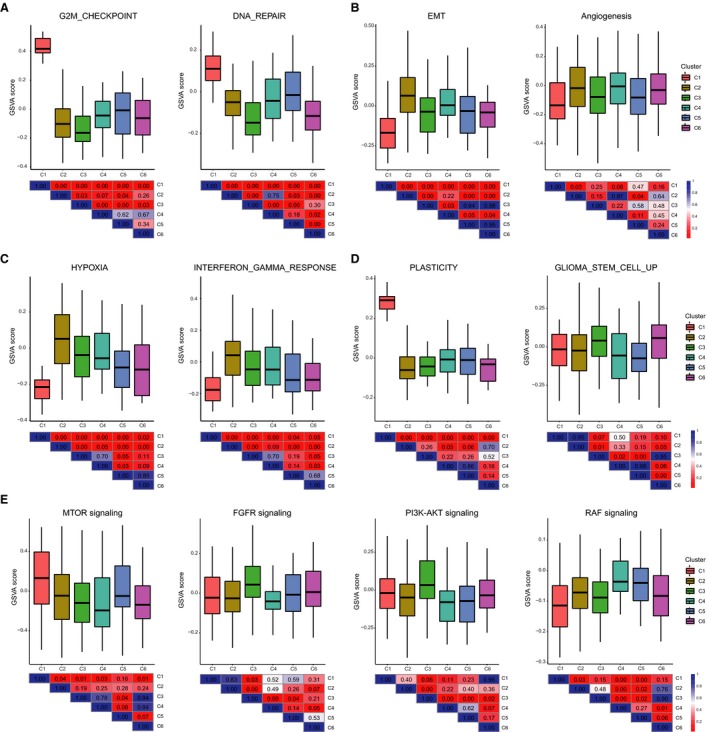
The status characterization of cell clusters. Boxplots showing the GSVA scores of each cell cluster for cell cycle‐associated signatures (A), invasion‐ and metastasis‐associated signatures (B, C), GBM cell plasticity and GSC signatures (D), and key signaling pathway genes (E). The diverse signatures and pathway genes were extracted from MSigDB. Heatmap in the bottom depicting the *P* value by Wilcoxon rank‐sum test for comparing each pair of clusters.

Distinct transcriptional profiles between cell clusters suggested divergent tumor cell behavior. We next used the predefined gene sets to estimate and compare the cancer hallmark‐associated status between clusters, in which Wilcoxon rank‐sum test was used to calculate the statistical significance. We found that cluster 1 was strongly associated with G2M checkpoint and DNA repair, both of which control the cell cycle (Fig. 2A). To confirm this, we used validated gene signatures previously shown to identify G1/S and G2/M cell cycle phases and distinguishing high cycling from low cycling cells to determine their cell cycle status. Compared with other clusters, cluster 1 showed higher expressions of G1/S and G2/M signatures. All cells in cluster 1 were cycling cells (see Materials and methods) and *MKI67* exclusively expressed in this cluster (Fig. S1B–D), suggesting their high proliferative activity. Cluster 2 showed relatively higher expression of EMT and angiogenesis‐associated genes (Fig. 2B), implying it may contain tumor cells with invasive potential. Consistently, it was also enriched for hypoxia‐ and inflammation‐associated genes (Fig. 2C), both of which can induce cancer cell migration and promote cancer progression (Bald *et al.*, 2014; Joseph *et al.*, 2015).

We then obtained glioma‐related signatures to investigate cell clusters. Compared with cluster 1, cluster 6 demonstrated lower expression of plasticity genes but relatively higher expression of stem cell‐related genes (Fig. 2D). Moreover, we selected pathways closely involved in cancer progression to evaluate their activation status and revealed that mTOR signaling was enriched in cluster 1, cluster 3 highly expressed FGFR and PI3K‐AKT signaling‐related genes, while clusters 4 and 5 showed high RAF signaling pathway activity (Fig. 2E). These results indicated that different cell subpopulations in GBM displayed various status which reflect distinct tumor biology, providing novel insights into molecular signatures of GBM cell clusters, including both intrinsic properties and regulation of signaling pathways.

### 3.3 Branched structure of tumor cells reveals the ‘stem‐to‐invasion path’ in glioblastoma

**Figure 3:**
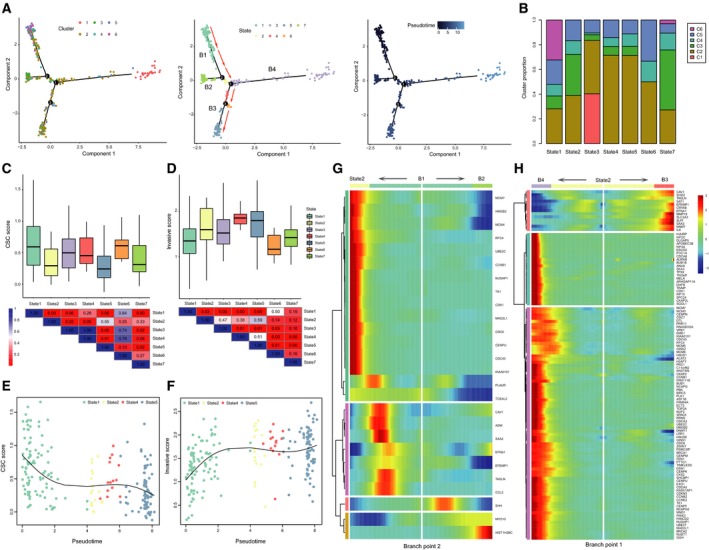
Reconstruction of a trajectory reveals branched structure and the stem‐to‐invasion path. (A) The single‐cell trajectory reconstructed by Monocle contains four main branches. Cells start at B1 and progress to B2 through branch points 2. Cells travel to B3 or B4 through branch point 1. Cells are colored based on cluster (left), state (middle), and pseudotime (right). Red arrows indicate the defined ‘stem‐to‐invasion path’. (B) The proportion of cells for each cluster in seven states defined by Monocle. Boxplot showing the CSC (C) and invasive (D) scores for each state with the *P* value shown as heatmap below. The CSC scores decrease (E) and the invasive scores increase (F) as a function of pseudotime in path that contains states 1, 2, 4, and 5 cells. A natural spline was used to model gene expression as a smooth, nonlinear function over pseudotime. (G) Heatmap depicting genes with a branch‐dependent manner for branch point 2. Each row represents the dynamic expression of a gene. The heatmap center represents the root of the trajectory, and proceeding to the left follows the kinetic curve from the root along the trajectory to state 2. Proceeding to the right yields the curve from the root to B2. (H) Heatmap depicting branch‐dependent genes for branch point 1. The heatmap center represents state 2, and proceeding to the left means to B4. Proceeding to the right means to B3.

Based on the observation that cluster 6 showed stem cell‐like signatures while clusters 1 and 2 displayed strong cell cycle activity and invasive potential, respectively, we speculated that single‐cell RNA‐seq may capture the main transformed processes of CSCs during tumor progression. To address this, Monocle was used to reconstruct a trajectory which mainly contained four branches (denoted ‘B1’, ‘B2’, ‘B3’, and ‘B4’) and grouped cells into seven states (Fig. 3A, see Materials and methods). Notably, the trajectory’s root (B1) was populated by the majority of cluster 6 cells (Fig. 3B), consistent with the functional annotation and status characterization of cluster 6. To confirm the stem cell‐like identity of cluster 6, we used the well‐known markers of GBM stem cell (GSCs; such as *SOX2*, *PROM1*/*CD133*, *SOX4*, *THY1*, and *ASCL1*) to define a CSC score for each cell (see Materials and methods). We found that the CSC scores were high in B1 (state 1; Fig. 3C), similar to the dynamic changes of these markers (Fig. S2A).

Most interestingly, B3 (refers to cells of states 4 and 5) was populated by cells from cluster 2 (Fig. 3B), implying the cells in this branch may acquire invasive potential. To validate this, we collected experimentally confirmed genes which could contribute to the invasion of GBM cells (such as *ZEB1*, *HNRNPC*, *WNT5A*, and *DRAM1*) to evaluate the invasive scores for each cell (see Materials and methods). As shown in Fig. 3D, cells in states 4 and 5 showed the highest invasive scores, followed by state 2 cells which located between two main branch points. This observation was supported by the high scores for cluster 2 cells and low scores for cells in clusters 5 and 6 (Fig. S2B). Based on the above findings, we considered that the cells travelled from B1 through branch point 2, state 2, and then to B3, representing the tumor progression from GSCs to invasive cells (denoted ‘stem‐to‐invasion path’), during which the CSC scores gradually decreased and invasive scores gradually increased as a function of pseudotime (Fig. 3E,F and Fig. S2C). The ‘stem‐to‐invasion paths’ were also identified when we performed the pseudotime analysis for each patient separately and similar change patterns of CSC and invasive scores along these paths were observed in most patients except for MGH29 (Figs S3–S6).

In order to further explore our assumption, we first identified 26 and 110 genes with branch‐dependent expression (false discovery rate < 1e‐4, see Materials and methods) for branch points 2 and 1, respectively (Fig. 3G,H). Cells traveling from B1 to state 2 highly expressed genes involved in both cell cycle and tumor invasion at the later stage (Fig. 3G), which was accordant with its mixed status facing the selection of two paths (B3 or B4). Among those genes, *CAV1*, a principal structural component of caveolar membrane domains, has been widely reported to promote tumor invasion and metastasis (Huang *et al.*, 2012; Joshi *et al.*, 2008). There is also evidence for association of *SAA2* (serum amyloid A2) with invasiveness of glioma cells (Knebel *et al.*, 2013). Notably, the expression of both genes further increased when cells travelled to B3 from state 2 (Fig. 3H). During this process, genes such as *SOD2*, *MMP19*, *SLC2A3*, and *SLPI* were also upregulated, all of which were related to the invasion and migration of tumor cells (Mikami *et al.*, 2016; Muller *et al.*, 2010; Ren *et al.*, 2017).

**Figure 4:**
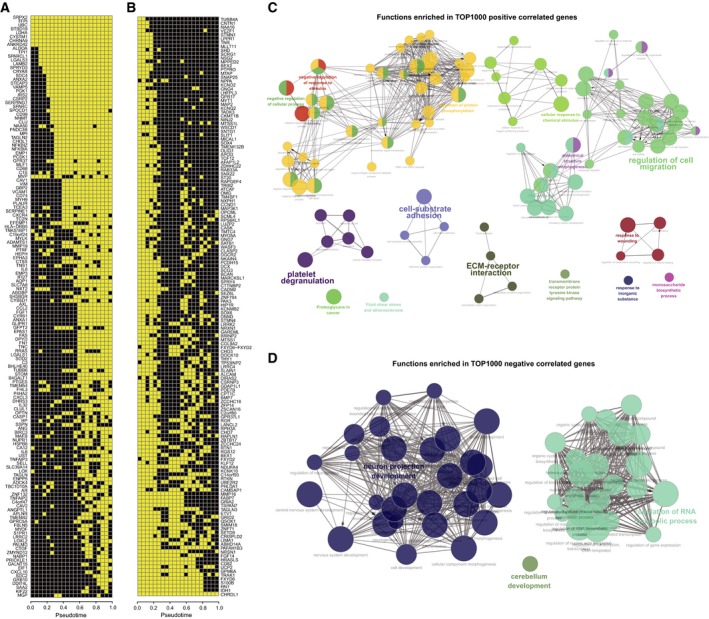
Dynamic molecules underlying tumor cell progression. On (yellow) or off (black) binary states of top 150 positively correlated genes (A) and negatively correlated genes (B). Functional annotations for top 1000 positively (C) and negatively (D) correlated genes are implemented by ClueGO.

Next, we sought to delineate the molecular events underlying the stem‐to‐invasion path. We generated a list of the top 1000 positively correlated genes with pseudotime along this path (Spearman correlation coefficient > 0.28), as well as the top 1000 negatively correlated genes (Spearman correlation coefficient < −0.22). A HMM was used to determine the binary on/high or off/low expression state of each gene along pseudotime with an unbiased fashion (see Materials and methods). Plots of the top 150 genes each from the two lists showed distinct but sequential transition patterns of gene expression (Fig. 4A,B). To obtain biological insights into these dynamic processes, we performed gene ontology analysis using ClueGO (Bindea *et al.*, 2009). Positively correlated genes revealed enrichment for invasion‐associated processes such as cell migration, cell adhesion, and ECM–receptor interaction (Fig. 4C), while negatively correlated genes enriched for neuron development and RNA metabolic process (Fig. 4D).

Taken together, these results demonstrated that the stem‐to‐invasion path can partially represent the transformed process from GBM stem‐like cell to invasive cells, and reflect the molecular cascades during GBM progression.

### 3.4 Extra data reproduces similar ‘stem‐to‐invasion path’ in glioblastoma progression

To validate whether the stem‐to‐invasion path could be recaptured in other GBM data, we obtained another single‐cell RNA‐seq data set of GBM (published by Darmanis *et al. *(2017) which contains 3589 cells from four patients. Following the same data processing, the final number of tumor cells for validation was 882.

**Figure 5:**
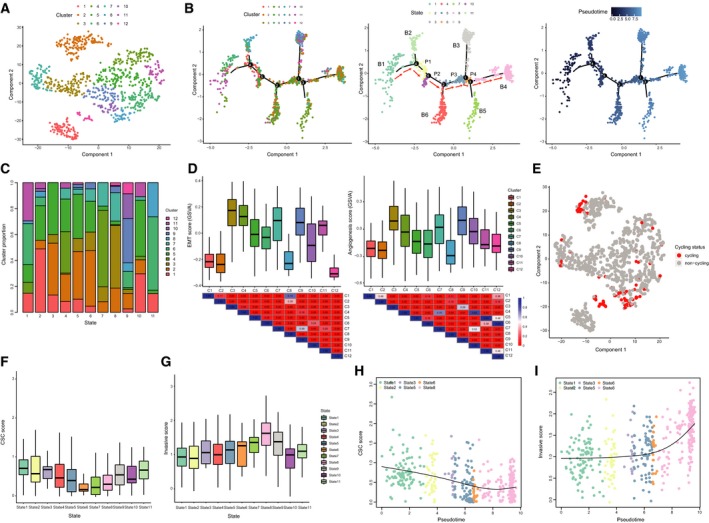
Validation using an independent single‐cell GBM data. (A) T‐SNE plot of tumor cells showing 12 clusters. (B) The single‐cell trajectory reconstructed by Monocle contains six main branches. Cells travel from the root B1 to B4 through P1‐P4. Cells are colored based on cluster (left), state (middle), and pseudotime (right). Red arrows indicate the defined ‘stem‐to‐invasion path’. (C) The proportion of cells for each cluster in 11 states. (D) Boxplots showing the GSVA scores of each cluster for EMT‐ and angiogenesis‐associated signatures with *P* value shown as heatmap below. (E) The cycling status of tumor cells. Red points represent identified cycling cells, and gray points represent noncycling cells. Boxplots showing the CSC (F) and invasive (G) scores for each state. The CSC scores decrease (H) and the invasive scores increase (I) as a function of pseudotime in path that contains cells of states 1, 2, 3, 5, 6, and 8.

In validation data, we identified 12 clusters (Fig. 5A). Then, a trajectory was reconstructed by Monocle, which contained six main branches (denoted ‘B1’ to ‘B6’) and four paths between branch points (denoted ‘P1’ to ‘P4’), which grouped cells into eleven states (Fig. 5B). We found B1 (state 1) contained most of cluster 11 cells and part of cluster 6 cells, B3 (state 9) contained most of cells in clusters 9, 10, and 12, while most of cluster 3 and 7 cells located in B4 (Fig. 5C). To determine whether this trajectory had similar structure with that in data from Patel *et al*., we first evaluated the expression of EMT‐ and angiogenesis‐associated genes, which showed that clusters 3, 4, 7, and 9 have higher scores (Fig. 5D). Notably, cells in these clusters mainly enriched in B4, corresponding to state 8, which consistently displayed the highest invasive score with an incremental pattern (Fig. 5G). Next, we detected the expression patterns of G1/S and G2/M signatures and found their enrichment in clusters 8, 10, and 12, corresponding to B3, consistent with the enrichment of cycling cells (Fig. 5E and Fig. S7A,B). Notably, compared with state 8 (B4), state 1(B1) showed higher CSC scores (Fig. 5F), and similar to data from Patel *et al*., the CSC scores gradually decreased as pseudotime increased for all cells (Fig. S7C). Therefore, we considered the path where cells travelled from the root B1 through P1, P2, P3, and P4 and finally to B4 as the ‘stem‐to‐invasion path’ here (Fig. 5B). As expected, the CSC scores were also decreased and the invasive scores were gradually upregulated as a function of pseudotime along this path, with an accelerating climb in the late stage (Fig. 5H,I). Similar functions were also enriched by top correlated genes (Fig. S7D–G). These results suggested that we observed similar branched structure and further recaptured the ‘stem‐to‐invasion path’ in an independent single‐cell data of GBM.

### 3.5 Identify crucial factors involved in the acquisition of invasive potential

**Figure 6:**
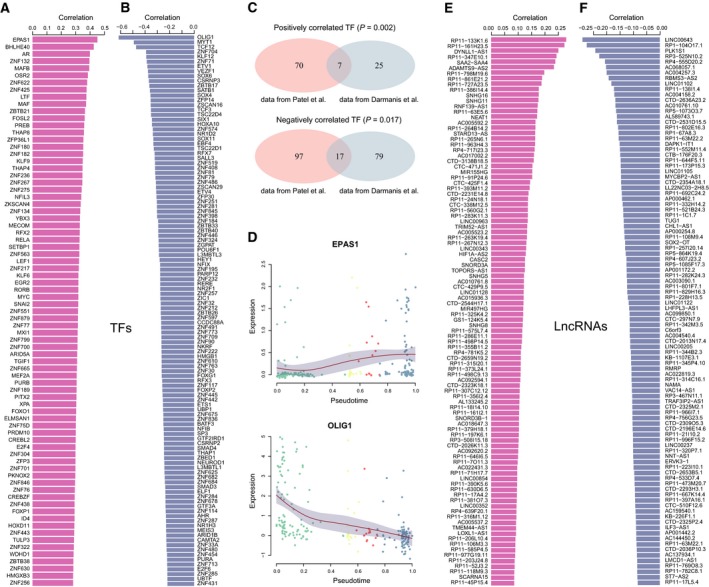
TFs and lncRNAs identified in data from Patel *et al*. (2014). List of upregulated (A) and downregulated (B) TFs as well as their Spearman correlation coefficient with pseudotime. (C) Venn diagrams showed the significant overlaps of TFs between both GBM data sets. *P* values were calculated by hypergeometric test. (D) Expression profiles of the most positively correlated TF EPAS1 and the most negatively correlated TF OLIG1. Data points are fitted with local polynomial regression fitting (red lines) with 95% confidence interval (gray area). Cells are colored based on their states. List of the top 100 upregulated (E) and downregulated (F) lncRNAs as well as their Spearman correlation coefficient with pseudotime in GBM1.

**Figure 7:**
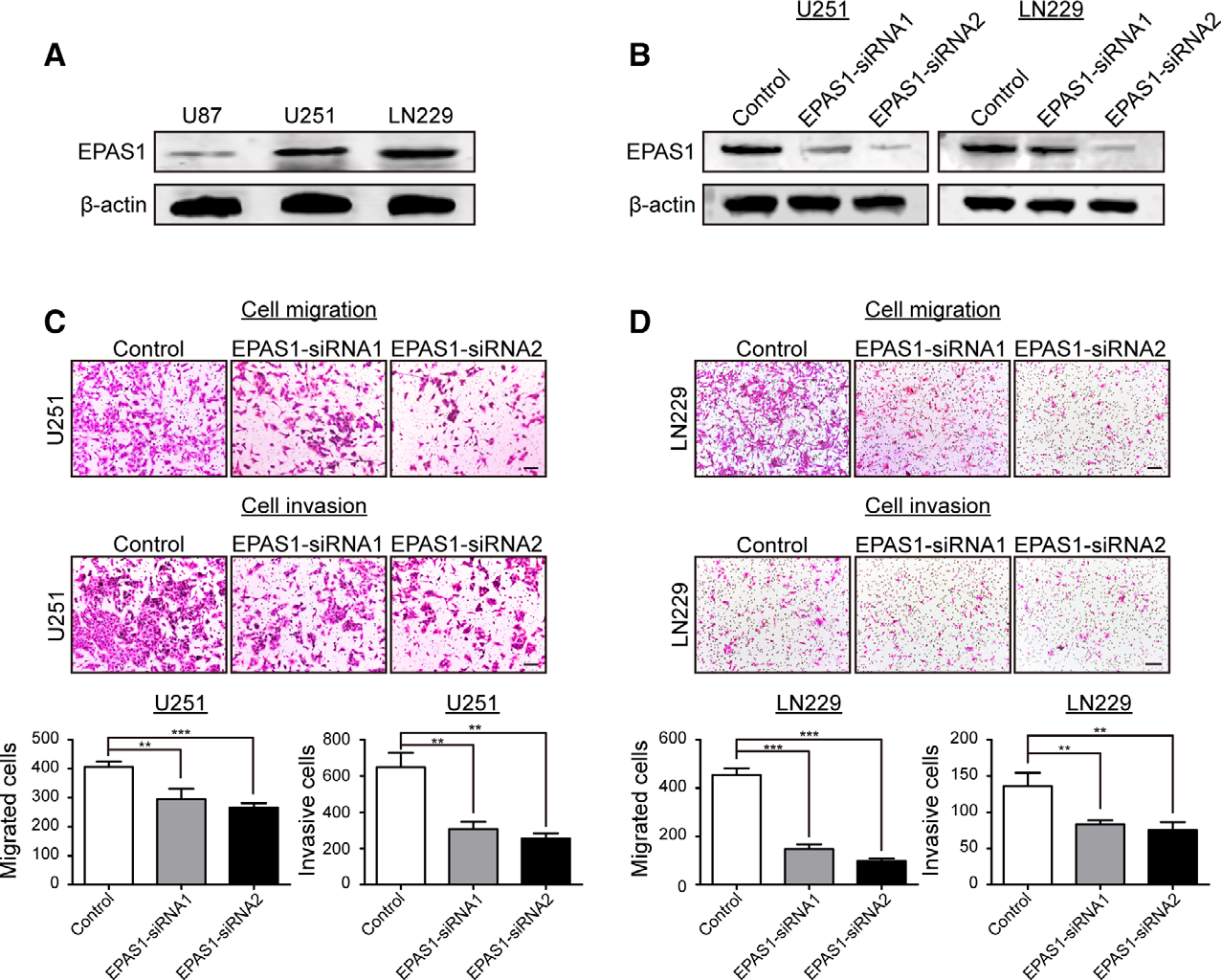
Knockdown of EPAS1 inhibited GBM cell migration and invasion *in vitro*. (A) Endogenous EPAS1 expression status in three GBM cell lines. (B) EPAS1 expression was efficiently knocked down by two targeted siRNAs (siRNA1 and siRNA2) in U251 cells and LN229 cells as detected by Western blotting. Silencing *EPAS1* expression suppressed cell migration and invasion capacity of U251 (C) and LN229 (D) cells in the Transwell migration and invasion assay (magnification 100×). Scale bars = 500 μm. Results were summarized as mean ± SD of three independent experiments (**P < 0.01; ***P < 0.001, independent Student’s *t* test).

Given the stepwise changes of genes observed in the trajectory analysis, we sought to identify the molecules that drive the acquisition of invasive potential and further promote the ‘stem‐to‐invasion’ progression. We first focused on transcription factors (TFs) in the two lists of top correlated genes identified above, which were obtained from AnimalTFDB (Zhang *et al.*, 2012). A total of 77 upregulated and 144 downregulated TFs were extracted in data from Patel *et al*. (Fig. 6A,B). Similarly, we also screened 32 upregulated and 96 downregulated TFs in data from Darmanis *et al*. (Fig. S8A,B). Notably, there were significant overlaps of these two sets of TFs between both data sets (Fig. 6C and Table S3). Interestingly, *EPAS1* was the most positively correlated TF shared in both data sets (Fig. 6D and Fig. S8C). Although some reports correlated it with tumor biology (Cruzeiro *et al.*, 2018), few studies revealed its role in the invasion of GBM cells. On the contrary, many of the top downregulated TFs control cell cycle and stemness, such as *MYT1*, *SOX6*, and *SOX4*, among which *OLIG1* was the top one in shared TFs. These accordant findings between both data sets indicated that these TFs, especially *EPAS1*, may indeed play crucial roles in controlling the invasive potential of GBM cells. To further validate the impact of EPAS1 on GBM cell invasiveness *in vitro*, we first analyzed endogenous EPAS1 expression in a panel of GBM cell lines (U87, U251, and LN229) by Western blotting (Fig. 7A). U251 and LN229 cell lines showed relative higher endogenous EPAS1 expression and therefore were selected for the knockdown study. The *EPAS1* gene was silenced by RNA interference using two targeted siRNAs (siRNA1 and siRNA2) in U251 and LN229. Both siRNAs could efficiently knock down *EPAS1* in GBM cells (Fig. 7B). Further, migration and Matrigel invasion assays also demonstrated that knockdown of EPAS1 significantly reduced the migration and invasion potential of both U251 and LN229 cell lines (*P* < 0.01, Student’s *t* test, Fig. 7C,D) as compared to control cells. Collectively, these results provided evidence that *EPAS1* silencing could inhibit GBM cell migrative and invasive capacity *in vitro*.

Previous studies have revealed the close relationship between lncRNAs and tumor progression (Li *et al.*, 2016). At single‐cell level, we found that lncRNAs had much more variable expression as shown by the high coefficient of variation for averaged expression than PCGs (Fig. S9A,B), suggesting their functional relevance. Thus, we calculated the correlations between lncRNA expression and pseudotime along the ‘stem‐to‐invasion path’ in both data sets of GBM. Among the top 100 correlated lncRNAs (Fig. 6E,F, Fig. S8D,E), we identified seven upregulated and six downregulated lncRNAs shared by both data sets (Table S4). For example, one of the positively correlated lncRNA SNHG16 showed significantly higher expression levels in GBM compared with normal brain cells (Fig. S9C), although its cell proportion was lower (Fig. S9D). Here, besides studies reporting the roles of SNHG16 in glioma tumorigenesis (Lu *et al.*, 2018; Mastrangelo *et al.*, 2018), we provided more evidence of SNHG16 as oncogene to promote GBM invasion. Moreover, most of the top upregulated lncRNAs showed significantly higher expressions in GBM than those in normal cells. These results indicated that lncRNAs may also play important roles in controlling the invasive potential of GBM cells.

## 4 Discussion

Ninety percent of solid tumor‐associated deaths have been attributed to the invasion and metastatic dissemination of cancer cells, including GBM. Therefore, we utilized scRNA‐seq data to explore the molecular cascades during GBM invasive progression at a high resolution, which provided new insights into the mechanism underlying the achievement of invasive potential of GBM cells.

Glioblastoma comprises morphologically and phenotypically diverse cells (Singh *et al.*, 2004), which promoted us to presume the existence of cells with high invasive potential, combining the fact of early occurrence of EMT in many cancer types. We indeed found that a group of cells showed high invasive scores. Interestingly, there seems to be a mutually exclusive pattern between G1/S scores and EMT scores, which were observed in both data sets of GBM (Fig. S10). That is, cells with high proliferative activity tend not to have invasive potential, and vice versa, which is supported by the observation that cells with high proliferative activity and those with high invasive scores located in different branches of the trajectory (Figs 3A and 5B). Moreover, we also performed functional enrichment analysis for genes upregulated in B2 (state 11), B3 (state 9), B5 (state 7), and B6 (state 4) of the constructed trajectory in data from Darmanis *et al*. We found that B2‐ and B3‐enriched genes were associated with glial cell differentiation, metabolic process, and cell cycle (Fig. S11). B5‐enriched genes were mainly involved in response to stress, regulation of cell motility, cell death, and protein location. B6‐enriched genes were associated with cell differentiation, cell proliferation, and mRNA catabolic process. These results indicate that different mechanisms may determine cells progressing to distinct outcomes during GBM progression.

The strength of scRNA‐seq derives from its high resolution. Here, we introduced the pseudotime method to capture and dissect transcriptional changes in cells along GBM progression, since we considered it as a continuous and heterogeneous process involving cancer initiation, proliferation, invasion, and metastasis (Hanahan and Weinberg, 2011). We identified a trajectory with branched structure in data from Patel *et al*. One branch represents the root of the trajectory, which showed relatively high CSC scores. Another two branches enriched proliferative cells and those with high invasive potential, respectively. These results are consistent with the complexity and heterogeneity of tumor progression. Further, we determined a path (the stem‐to‐invasion path) along which cells travelled from the root to the invasive branch, representing the progression of GSCs transforming to invasive cells. Consistently, the CSC scores gradually decreased while the invasive scores gradually increased during this process. Branch‐dependent expression analysis found that many known invasion‐associated genes, such as *CAV1*, *MMP19*, and *SLC2A3*, also showed gradual increase of expression. Notably, we discovered a similar branched trajectory as well as the ‘stem‐to‐invasion path’ in another validation data. The difference is that the trajectory in validation data has more complex structures because of the more number of tumor cells. Moreover, we expanded our research into the data of oligodendroglioma published by Tirosh *et al. *(2016b). We identified 11 clusters and constructed a trajectory containing five states (Fig. S12A,B). The CSC and invasive scores of cells showed a few differences across different states, although cells of states 1 and 2 showed significantly higher CSC scores, while cells of state 5 showed significantly higher invasive scores (Fig. S12C,D). Along the defined ‘stem‐to‐invasion path’ (defined as cells traveling from state 1 through state 2, state 3, and then to state 5), the CSC scores did not exhibit obvious differences, while the invasive scores present a weak increasing trend (Fig. S12E,F). Since these cells were taken from grade II oligodendrogliomas at early stage of clinical progression, we considered that in the initial steps of gliomagenesis, cells have not obtained evident potential to invade the surrounding tissues. All these observations made us believe that the ‘stem‐to‐invasion path’ identified in GBM data could veritably reflect the molecular events underlying GBM progression and help to identify the molecular determinants of invasion.

Therefore, we further identified the top correlated TFs, among which *EPAS1* (HLF2A, hypoxia‐inducible factor 2A) was the first and second TF ordered by correlation coefficients in both GBM data sets. Although previous studies have mentioned its involvement in brain tumors such as neuroblastoma (Mohlin *et al.*, 2015) and GBM (Wang *et al.*, 2018), few studies focused on the roles of *EPAS1* in GBM invasion. Our analyses revealed its dynamic transcriptional pattern during GBM progression, providing new evidence and insight into the contribution of *EPAS1* to achievement of invasion potential of GBM cells. But the detailed mechanism needs further investigation of future studies. Besides *EPAS1*, we also identified other six common TFs, including *FOSL2*, *PREB*, *YBX3*, *RELA*, *KLF6*, and *MYC*. Notably, expect for *MYC*, few researches have reported the correlation of most of these TFs with GBM, especially invasion. Moreover, given the important roles of lncRNAs in tumor biology, we also identified the top correlated lncRNAs, which also need further investigation and validation of their functional mechanisms in GBM invasion.

## 5 Conclusions

In summary, our study used single‐cell RNA‐seq to provide a subtle delineation of cellular heterogeneity in GBM, reveal the invasive path, and determine key factors contributing to GBM invasion at a high resolution. The new insights into GBM progression may be useful for the clinical treatment, and the identified crucial factors may offer a selective and efficient therapeutic target for GBM, and possibly other solid malignant tumors.

## Conflict of interest

The authors declare no conflict of interest.

## Author contributions

LP designed the research; FHG, LYW, and MJC collected and preprocessed the data; BP, JYX, and JH performed bioinformatics analysis; BP and LP wrote the manuscript. All authors read and approved the final manuscript.

## Supporting information


**Fig. S1** **.** The cell cycle status of tumor cells.
**Fig. S2** **.** Single‐cell trajectory detection uncovers GBM progression.
**Fig. S3** **.** Construction of a trajectory and pseudotime analysis for MGH26.
**Fig. S4** **.** Construction of a trajectory and pseudotime analysis for MGH28.
**Fig. S5** **.** Construction of a trajectory and pseudotime analysis for MGH29.
**Fig. S6** **.** Construction of a trajectory and pseudotime analysis for MGH30.
**Fig. S7** **.** The validation in another data set from Darmanis *et al*.
**Fig. S8** **.** TFs and lncRNAs identified in data from Darmanis *et al*.
**Fig. S9** **.** Expression patterns of lncRNAs in data from Patel et al. and normal brain cells.
**Fig. S10** **.** The mutually exclusive patterns between G1/S scores and EMT scores.
**Fig. S11** **.** Functional annotations for significantly upregulated genes in each branch of the trajectory constructed in data from Darmanis *et al*.
**Fig. S12** **.** Re‐analysis of the oligodendroglioma data from Tirosh *et al*.

**Table S1** **.** Housekeeping genes and marker genes used in this study.

**Table S2** **.** Significantly upregulated genes in each cluster identified by SCDE.

**Table S3** **.** The overlaps of TFs between both GBM data sets.

**Table S4** **.** The overlaps of lncRNAs between both GBM data sets.
