# Supplementary material for: Single‐cell RNA‐seq reveals the invasive trajectory and molecular cascades underlying glioblastoma progression
Source: Mol Oncol. 2019 Sep 17;13(12):2588–603. doi: 10.1002/1878-0261.12569 (PMC6887585; doi:10.1002/1878-0261.12569)
Supplement: Supplementary file 1 — Fig. S1 . The cell cycle status of tumor cells. Fig. S2 . Single‐cell trajectory detection uncovers GBM progression. Fig. S3 . Construction of a trajectory and pseudotime analysis for MGH26. Fig. S4 . Construction of a trajectory and pseudotime analysis for MGH28. Fig. S5 . Construction of a trajectory and pseudotime analysis for MGH29. Fig. S6 . Construction of a trajectory and pseudotime analysis for MGH30. Fig. S7 . The validation in another data set from Darmanis et al. Fig. S8 . TFs and lncRNAs identified in data from Darmanis et al. Fig. S9 . Expression patterns of lncRNAs in data from Patel et al. and normal brain cells. Fig. S10 . The mutually exclusive patterns between G1/S scores and EMT scores. Fig. S11 . Functional annotations for significantly upregulated genes in each branch of the trajectory constructed in data from Darmanis et al. Fig. S12 . Re‐analysis of the oligodendroglioma data from Tirosh et al. [file MOL2-13-2588-s001.pdf]

Supplementary Figures

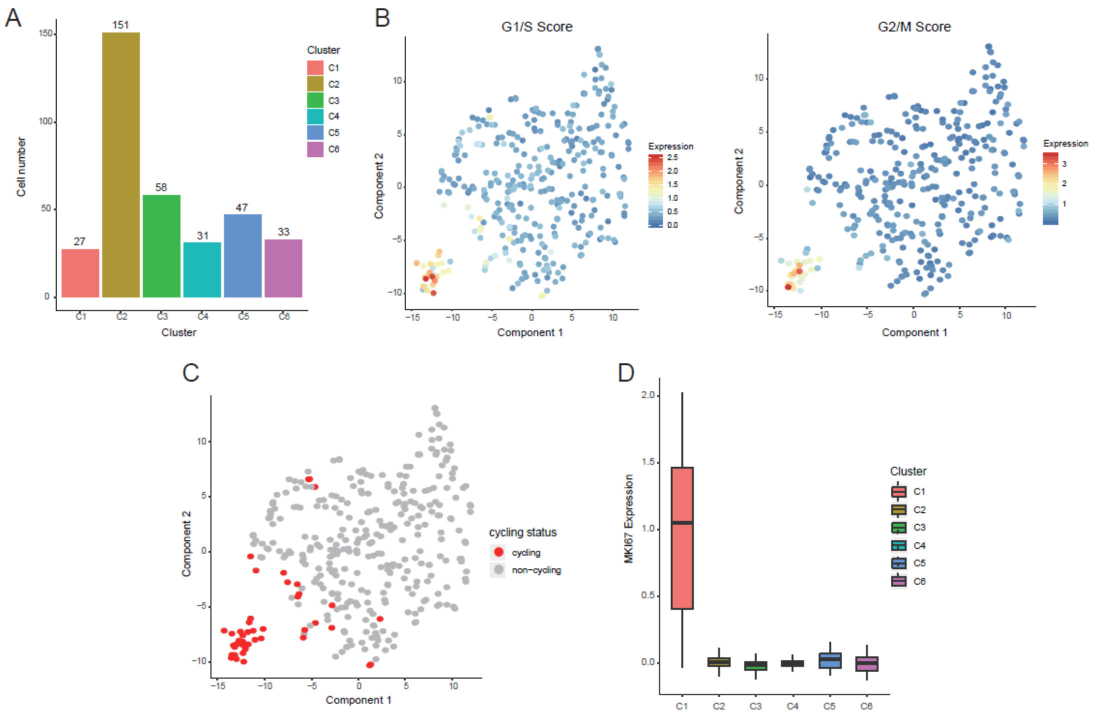

Figure S1. The cell cycle status of tumor cells. (A) The numbers of cell in each cluster. (B) T-SNE plot showing the G1/S and G2/M scores for each tumor cell. Cells are colored based on their scores, in which red represents high scores while blue represents low ones. (C) The cycling status of tumor cells. Red points represent identified cycling cells and gray points represent non-cycling cells. (D) Boxplot showing the *MKI67* expression levels in each cluster cells, which indicates that cluster 1 has significantly higher expression of *MKI67* compared to other clusters.

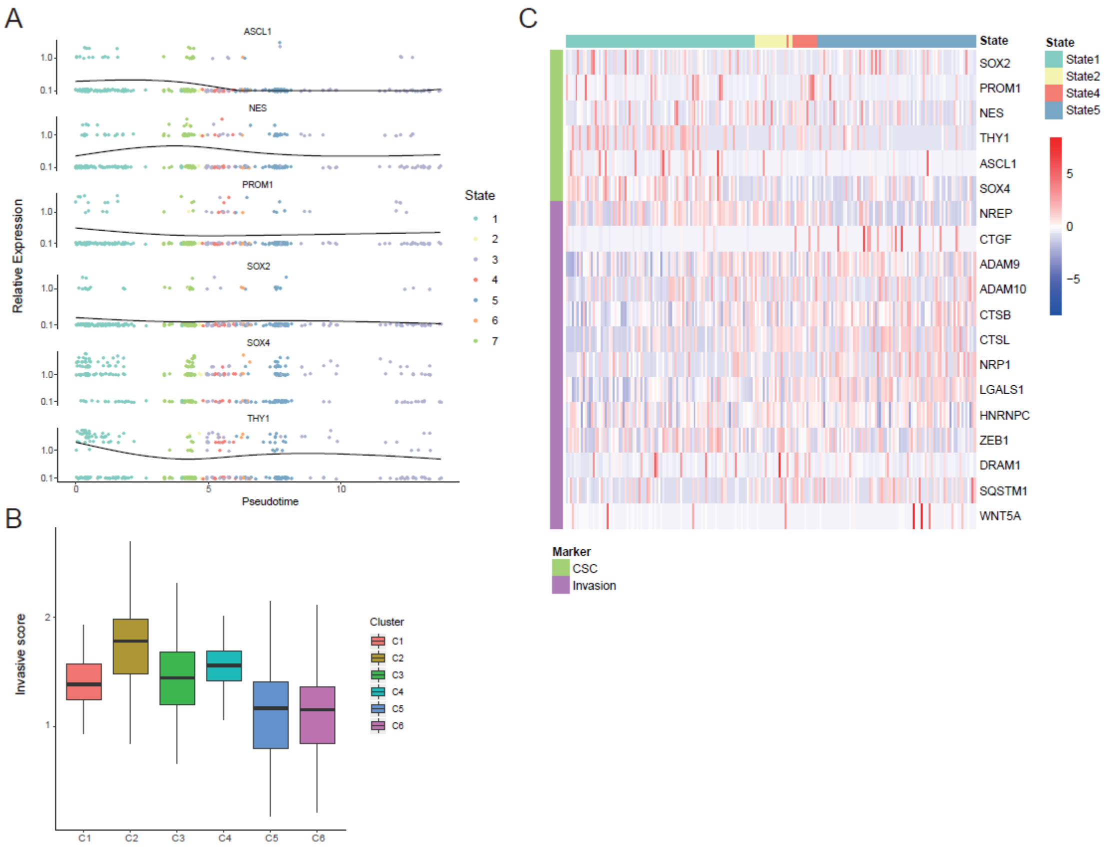

Figure S2. Single-cell trajectory detection uncovers GBM progression. (A) Kinetic curves for six glioma stem cell markers from the root to the end of the trajectory. Cells are colored based on state. (B) Boxplot showing the invasive scores in each cluster. (C) The heatmap showing the expression levels of CSC and invasion-associated markers along the “stem-to-invasion path”.

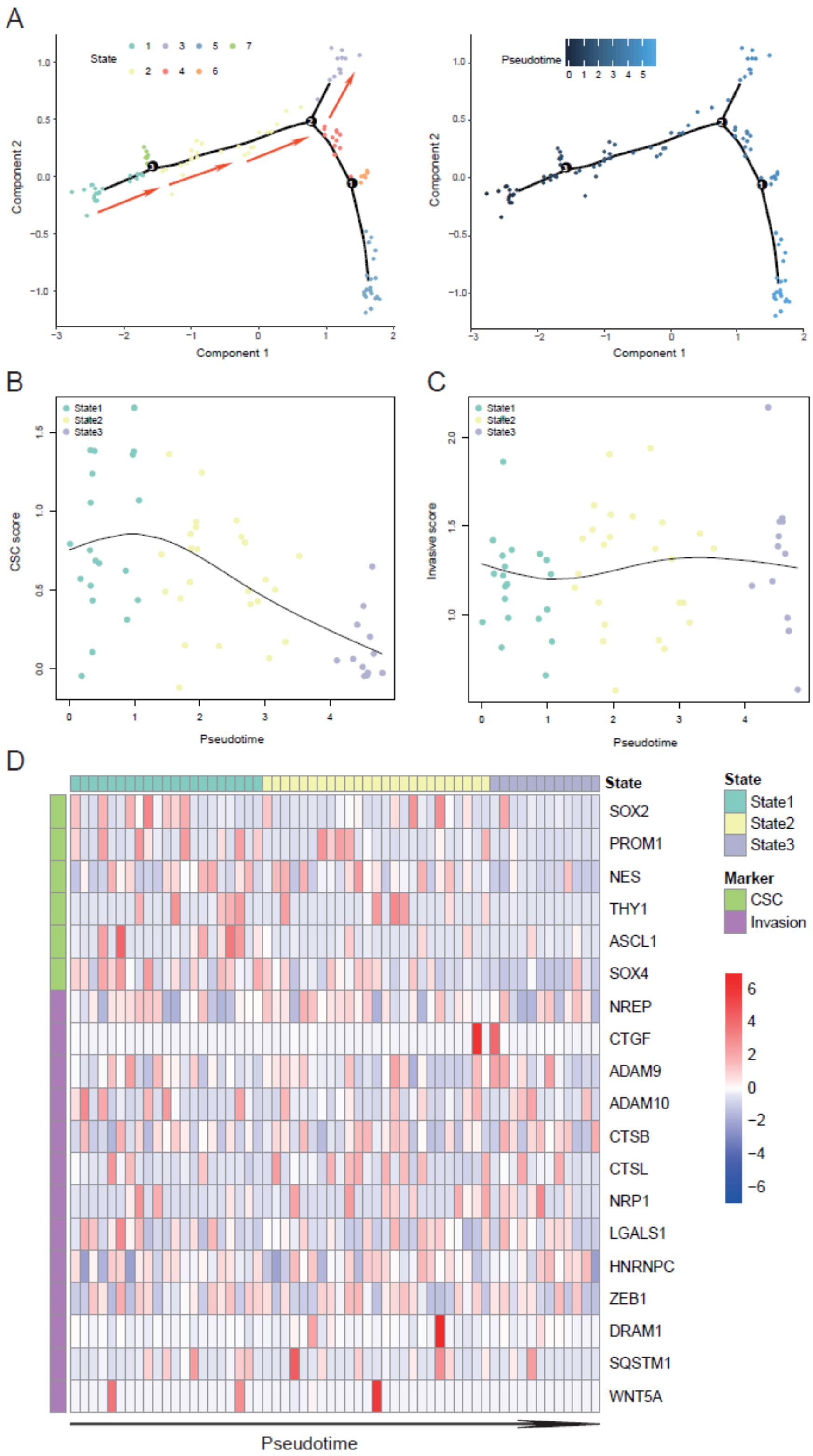

Figure S3. Construction of a trajectory and pseudotime analysis for MGH26. (A) The single-cell trajectory reconstructed by Monocle contains 7 states (left) and shows the pseudotime of each cell (right). Red arrows indicate the defined "stem-to-invasion path" (cells travel from state1 to state 3 through state 2). The scatter plots showing the changes of CSC scores (B) and invasive scores (C) along the path, respectively. (D) The heatmap showing the expression levels of CSC and invasion-associated markers along the path.

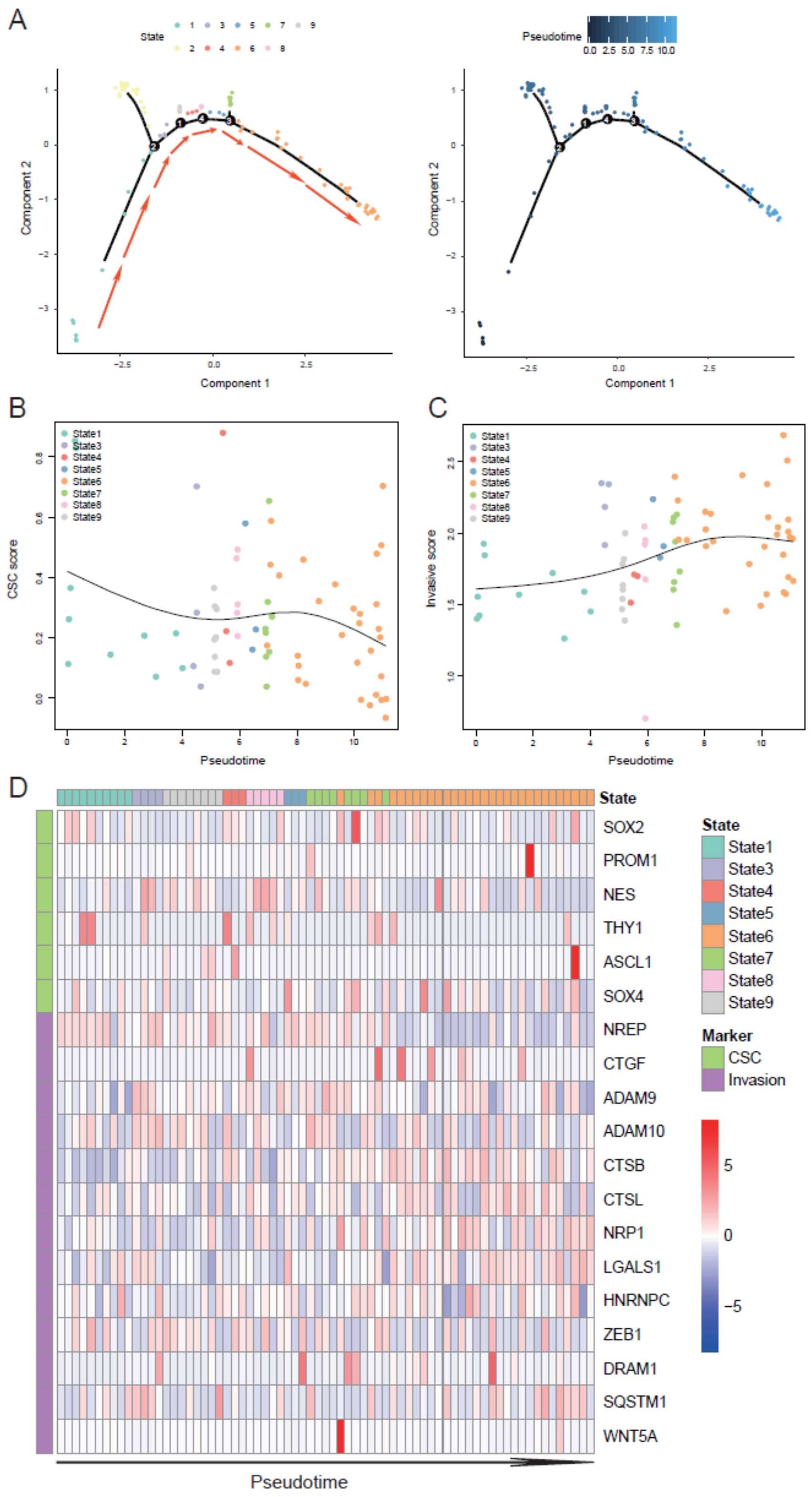

Figure S4. Construction of a trajectory and pseudotime analysis for MGH28. (A) The single-cell trajectory reconstructed by Monocle contains 9 states (left) and shows the pseudotime of each cell (right). Red arrows indicate the defined "stem-to-invasion path" (cells travel from state1 to state 9 without state 2). The scatter plots showing the changes of CSC scores (B) and invasive scores (C) along the path, respectively. (D) The heatmap showing the expression levels of CSC and invasion-associated markers along the path.

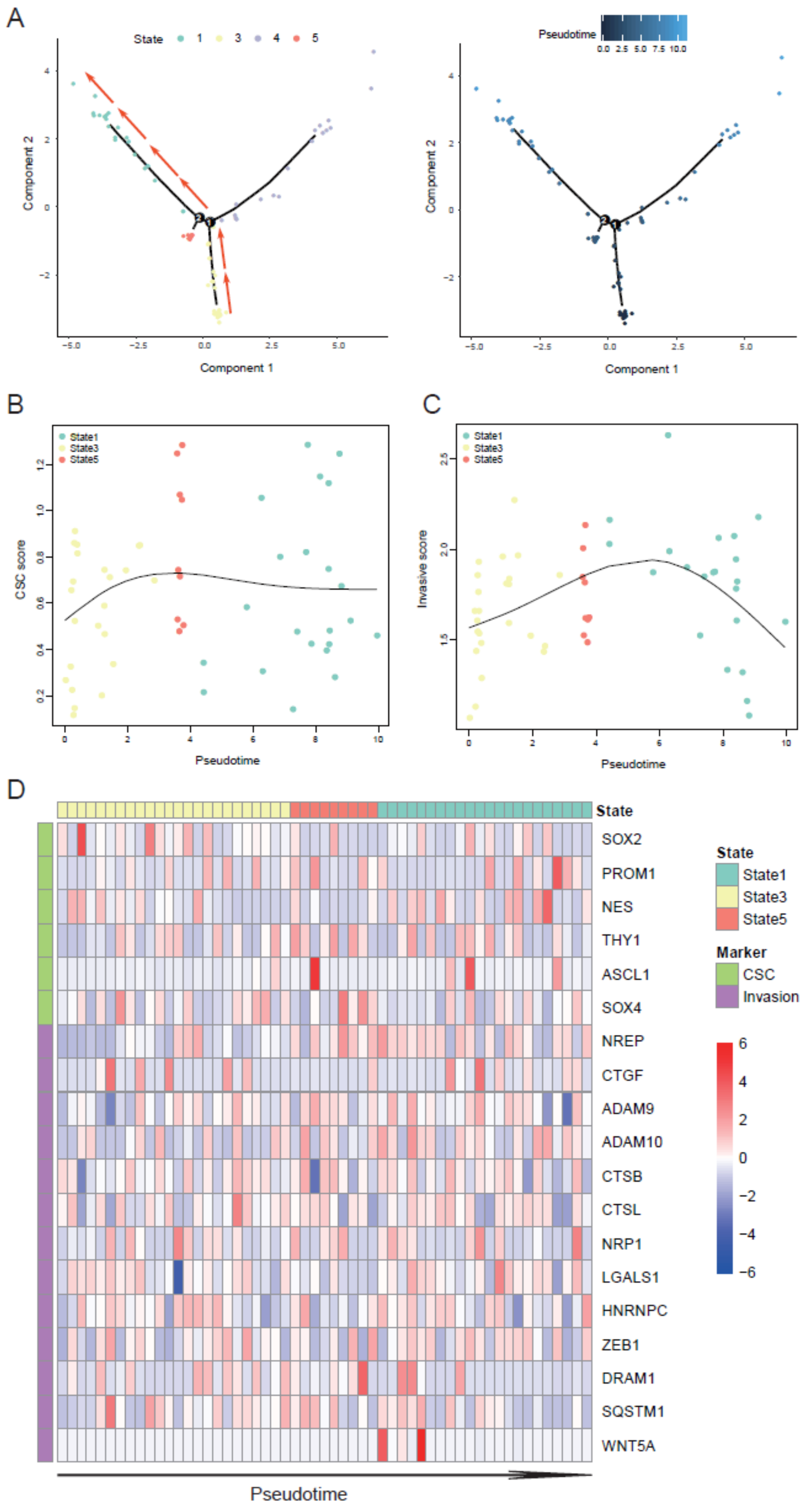

Figure S5. Construction of a trajectory and pseudotime analysis for MGH29. (A) The single-cell trajectory reconstructed by Monocle contains 4 states (left) and shows the pseudotime of each cell (right). Red arrows indicate the defined "stem-to-invasion path" (cells travel from state 3 to state 1 through state 5). The scatter plots showing the changes of CSC scores (B) and invasive scores (C) along the path, respectively. (D) The heatmap showing the expression levels of CSC and invasion-associated markers along the path.

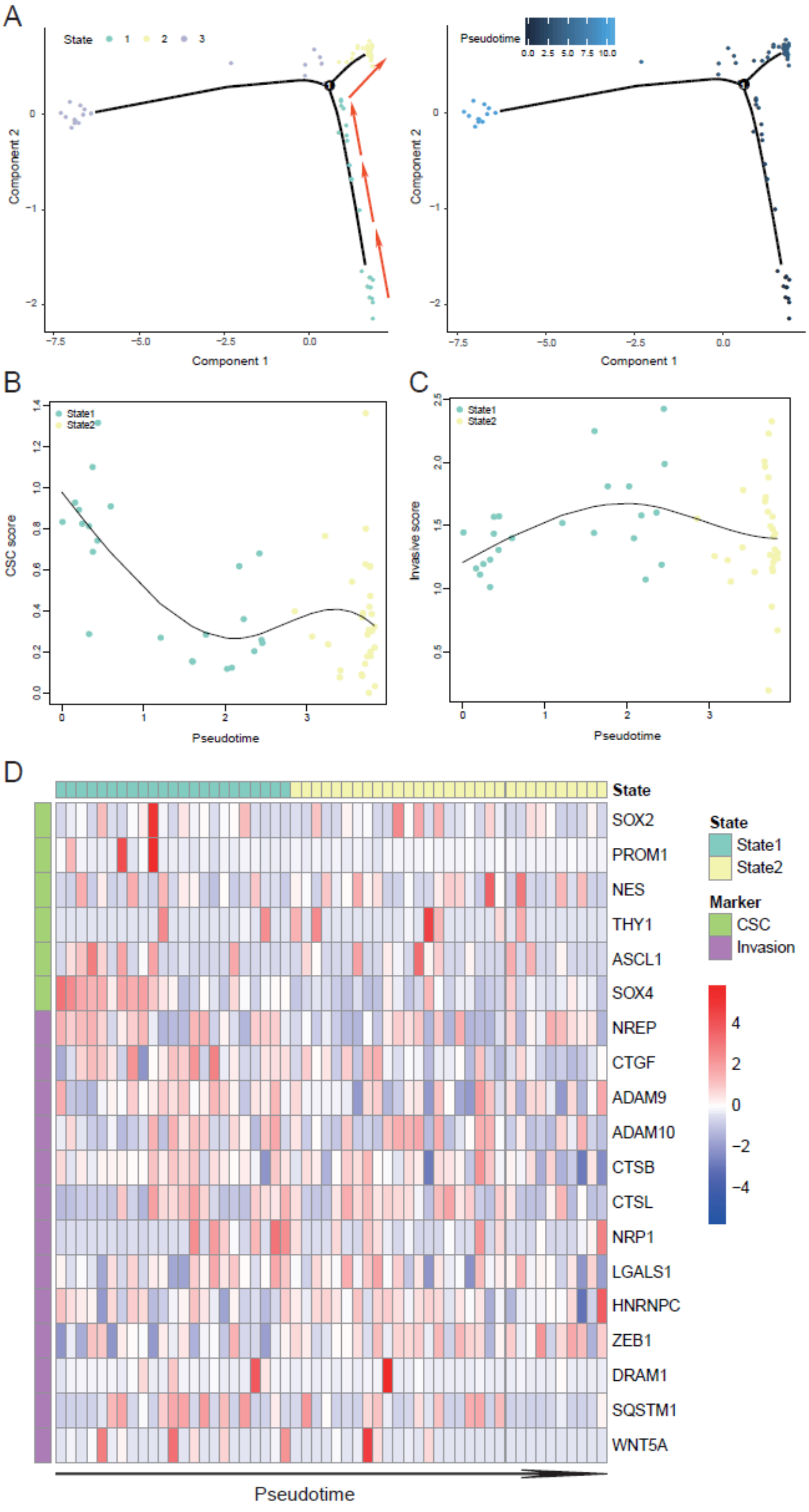

Figure S6. Construction of a trajectory and pseudotime analysis for MGH30. (A) The single-cell trajectory reconstructed by Monocle contains 3 states (left) and shows the pseudotime of each cell (right). Red arrows indicate the defined "stem-to-invasion path" (cells travel from state1 to state 2 without state 3). The scatter plots showing the changes of CSC scores (B) and invasive scores (C) along the path, respectively. (D) The heatmap showing the expression levels of CSC and invasion-associated markers along the path.

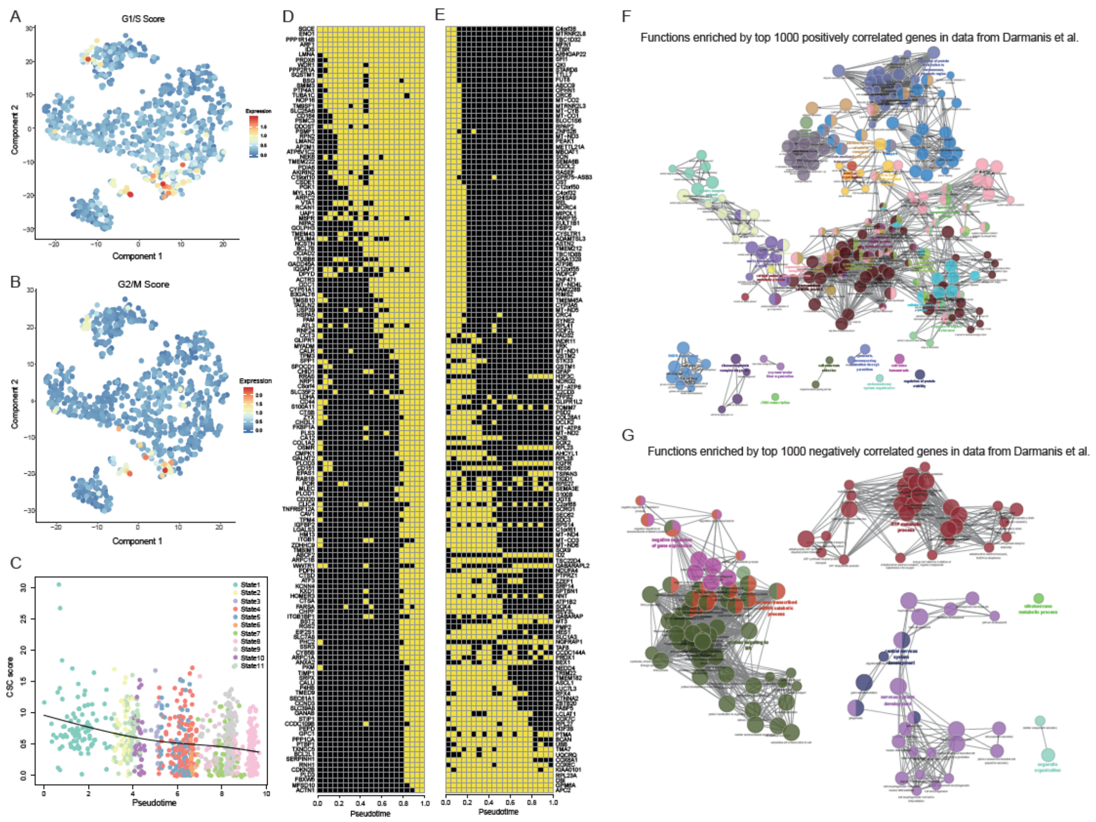

Figure S7. The validation in another data set from Darmanis et al. T-SNE plot showing the G1/S (A) and G2/M (B) scores for each tumor cell. Cells are colored based on their scores, in which red represents high scores while blue represents low ones. (C) The CSC scores decrease as pseudotime increased for all cells. On (yellow) or off (black) binary states of top 150 positively correlated genes (D) and negatively correlated genes (E). Functional annotations for top 1000 positively (F) and negatively (G) correlated genes are implemented by ClueGO.

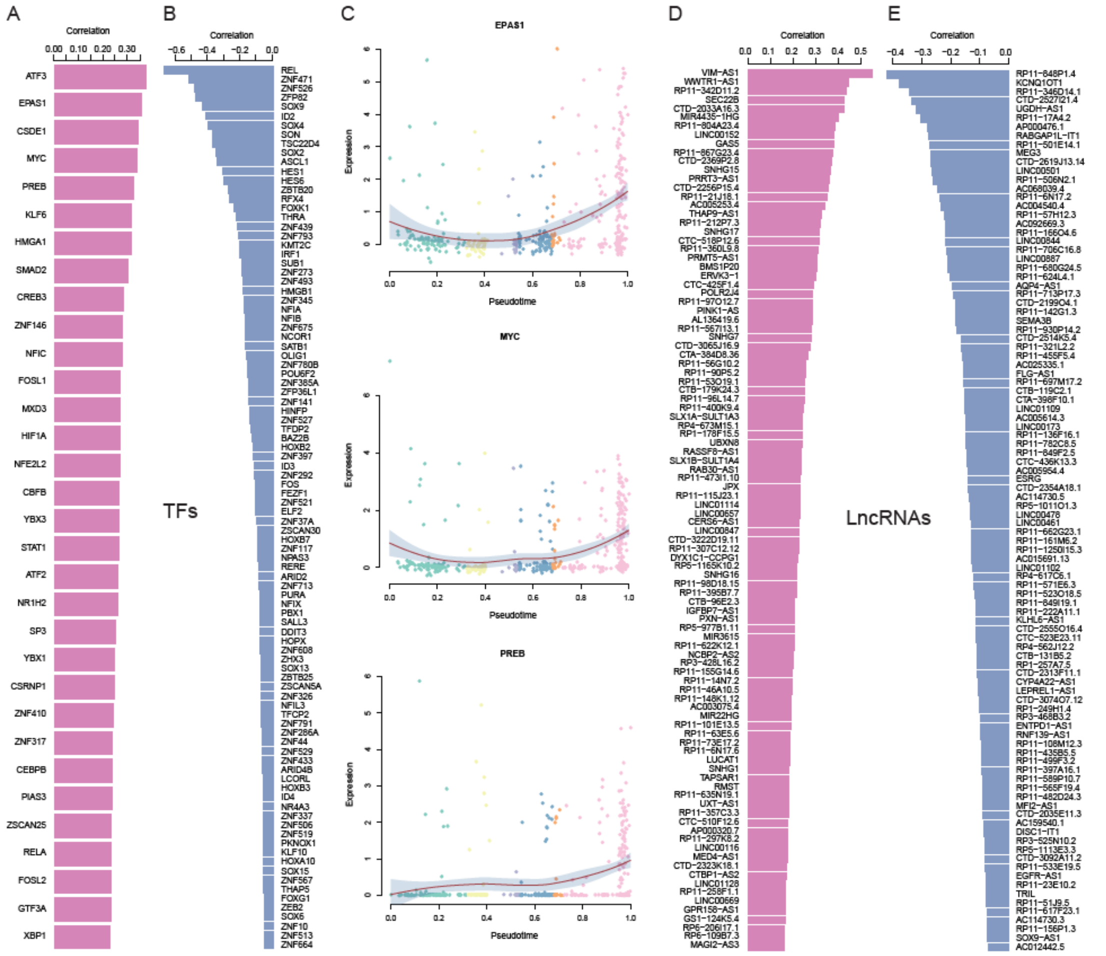

Figure S8. TFs and lncRNAs identified in data from Darmanis et al. List of upregulated (A) and downregulated (B) TFs as well as their Spearman correlation coefficient with pseudotime. (C) Expression profiles of examples for top regulated TFs including EPAS1, MYC and PREB along the invasive path, respectively. Data points are fitted with local polynomial regression fitting (red lines) with 95% confidence interval (gray area). Cells are colored based on their states. List of upregulated (D) and downregulated (E) lncRNAs as well as their Spearman correlation coefficient with pseudotime.

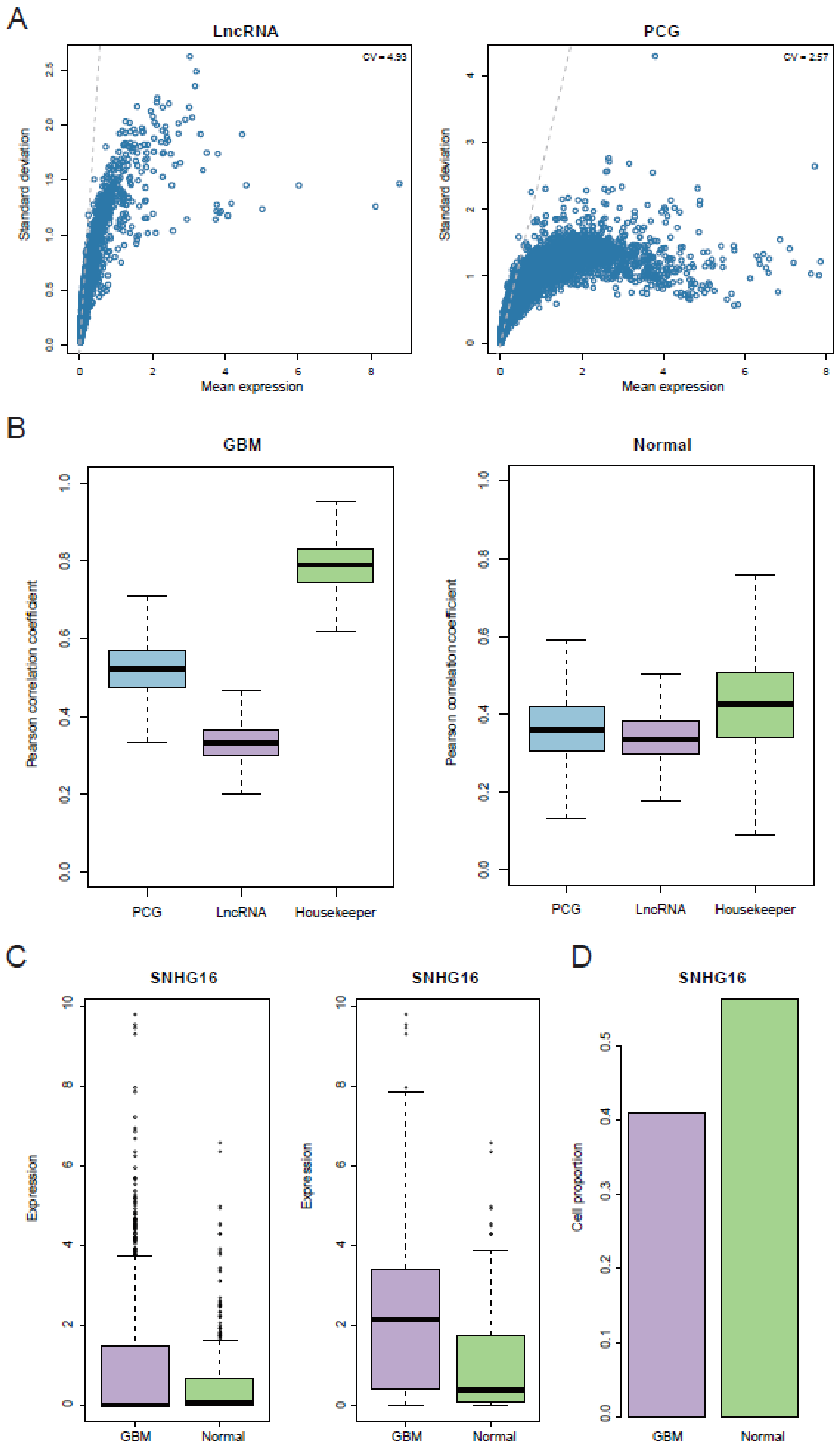

Figure S9. Expression patterns of lncRNAs in data from Patel et al. and normal brain cells. (A) Scatter plots evaluating the average expression levels of lncRNAs and PCGs with their variations across cells, respectively. (B) Comparison of correlation coefficients between cells based on lncRNAs, PCGs and housekeepers in GBM cells (left) and normal brain cells (right). (C) The expression levels of SNHG16 in all cells (left), expressed cells (middle) and its cell proportion (right) in data from Patel et al. and normal brain cells.

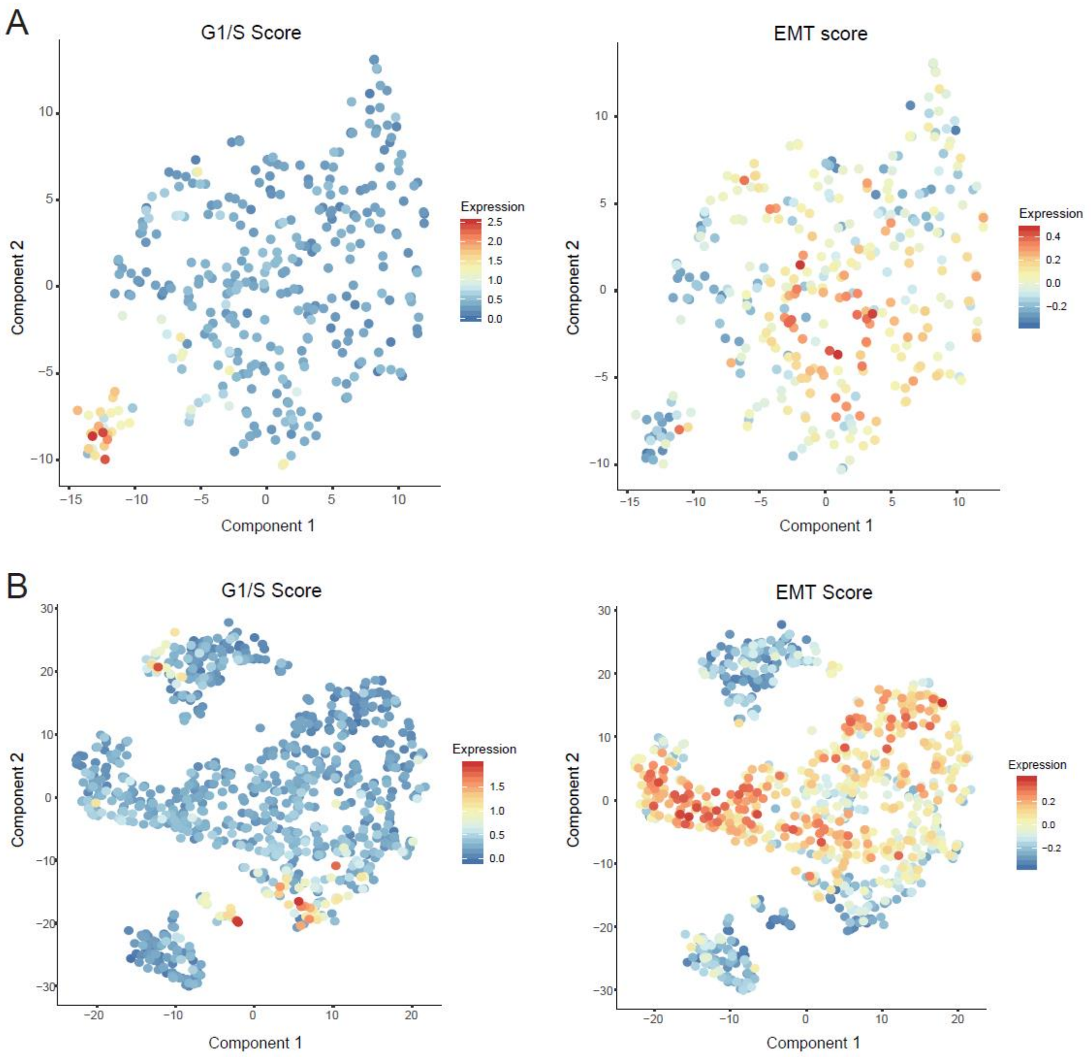

Figure S10. The mutually exclusive patterns between G1/S scores and EMT scores. (A) t-SNE plot showing the G1/S and G2/M scores for each tumor cell in data from Patel et al. Cells are colored based on their scores, in which red represents high scores while blue represents low ones. (B) t-SNE plot showing the G1/S and G2/M scores for each tumor cell in data from Darmanis et al.

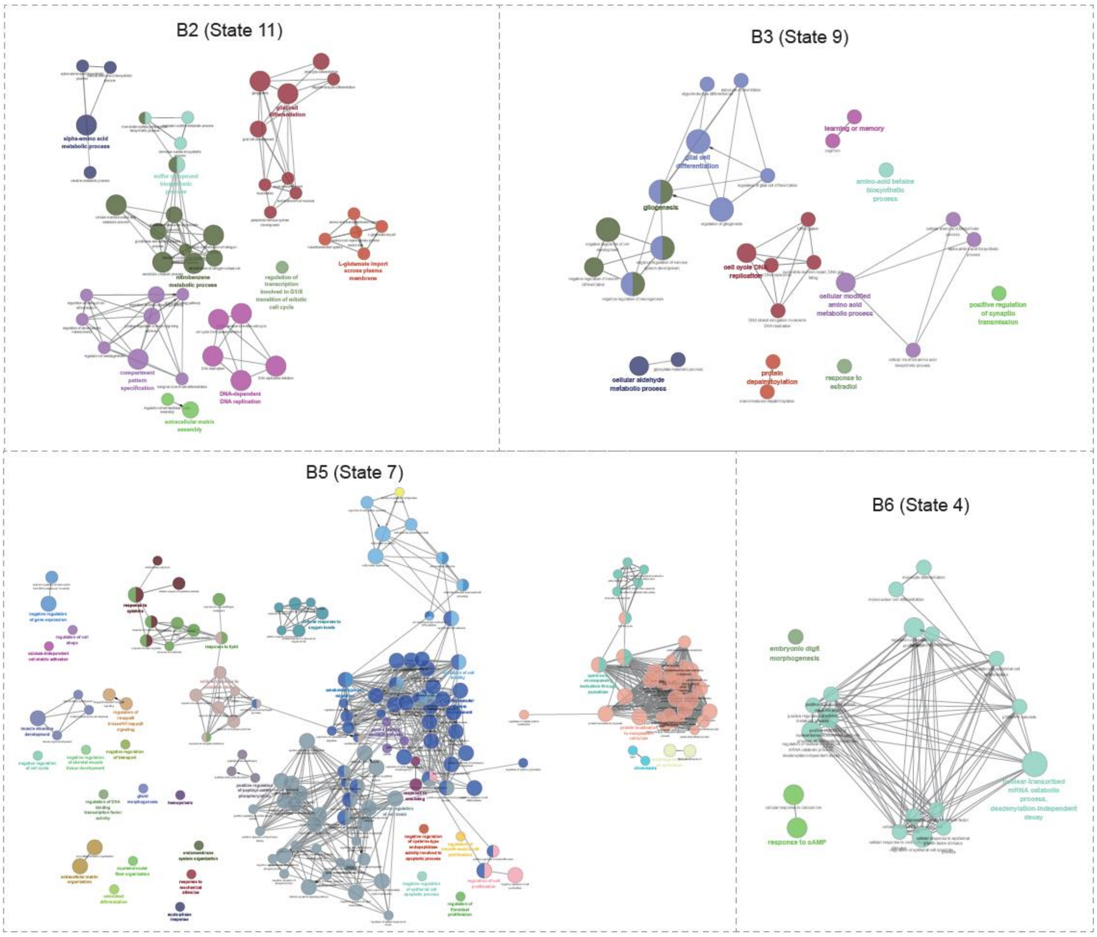

Figure S11. Functional annotations for significantly upregulated genes in each branch of the trajectory constructed in data from Darmanis et al. B2 and B3 enriched genes were associated with glial cell differentiation, metabolic process and cell cycle (Supplementary Figure S11). B5 enriched genes were mainly involved in response to stress, regulation of cell motility, cell death and protein location. B6 enriched genes were associated with cell differentiation, cell proliferation and mRNA catabolic process.

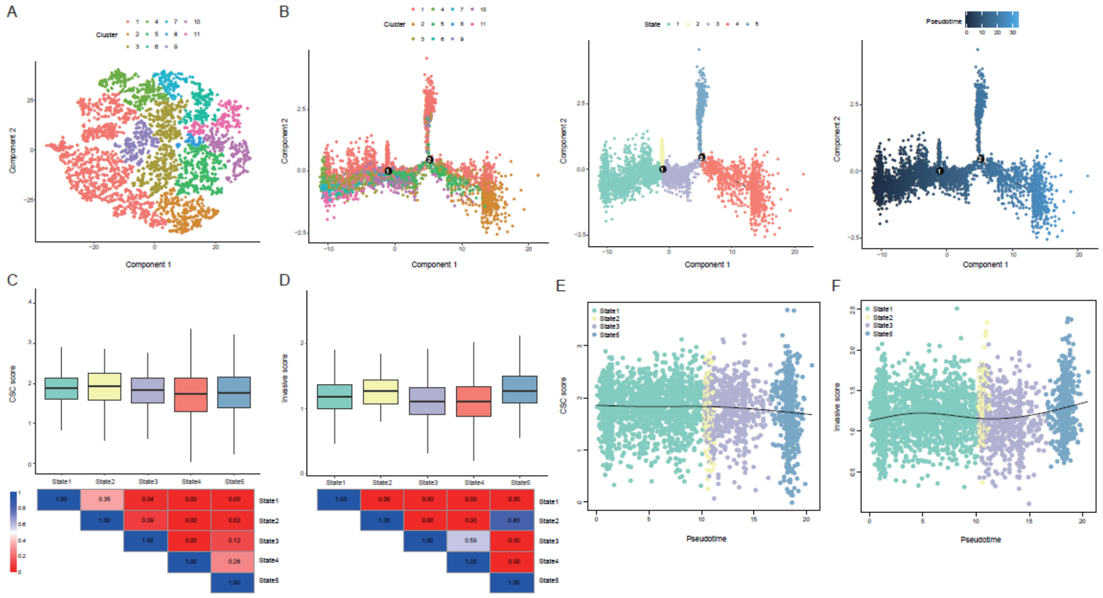

Figure S12. Re-analysis of the oligodendroglioma data from Tirosh et al. (A) t-SNE plot of tumor cells showing 11 clusters. (B) The single-cell trajectory is reconstructed by Monocle. Cells are colored based on cluster (left), state (middle) and pseudotime (right). The “stem-to-invasion path” is defined that cells travel from state 1 to state 5 without state 4. Boxplots showing the CSC (C) and invasive (D) scores for each state with the p value shown as heatmap below. The scatter plots showing the changes of CSC scores (E) and invasive scores (F) along the path, respectively.
